# Structure-symptom relationship with wide-area ultrasound scanning of knee osteoarthritis

**DOI:** 10.1038/srep44470

**Published:** 2017-03-15

**Authors:** Jana Podlipská, Juhani M. Koski, Päivi Kaukinen, Marianne Haapea, Osmo Tervonen, Jari P. Arokoski, Simo Saarakkala

**Affiliations:** 1 Research Unit of Medical Imaging, Physics and Technology, University of Oulu, Oulu, Finland; 2 Department of Internal Medicine, Mikkeli Central Hospital, Mikkeli, Finland; 3 Institute of Clinical Medicine, University of Eastern Finland, Kuopio, Kuopio, Finland; 4 Department of Physical and Rehabilitation Medicine, Kuopio University Hospital, Kuopio, Finland; 5 Department of Diagnostic Radiology, Oulu University Hospital, Oulu, Finland; 6 Medical Research Center, University of Oulu and Oulu University Hospital, Oulu, Finland; 7 Center for Life Course Health Research, University of Oulu, Oulu, Finland; 8 Department of Physical and Rehabilitation Medicine, University of Helsinki, Finland; 9 Helsinki University Hospital, Helsinki, Finland

## Abstract

The aetiology of knee pain in osteoarthritis (OA) is heterogeneous and its relationship with structural changes and function is unclear. Our goal was to determine the prevalence of wide-area scanned ultrasound-defined knee OA structural features and their association with pain and functional impairment in 79 symptomatic and 63 asymptomatic subjects. All subjects underwent ultrasound knee wide-area scanning and the severity of articular cartilage degeneration, the presence and size of osteophytes, and meniscal extrusion were evaluated. Subjects filled in a self-administrated questionnaire on present knee pain, and Western Ontario and McMaster Universities Osteoarthritis Index (WOMAC) on clinical symptoms and function over the past week. Cartilage changes were the most prevalent followed by lateral meniscal extrusion, osteophytes and medial meniscal extrusion. The global femoral cartilage grade associated strongly with pain and the WOMAC index. Site-specifically, early medial cartilage changes and thinning in sulcus and lateral site were associated with symptoms. The presence of femoral lateral osteophytes was also associated with both outcomes. Using the novel wide-area ultrasound scanning technique, we were able to confirm the negative impact of femoral cartilage OA changes on clinical symptoms. Presence, not necessarily size, of lateral femoral osteophytes was also associated with increased pain and disability.

The prevalence of osteoarthritis (OA) is rising worldwide with increasing age and obesity of the population^1^. In knee OA, pain is the hallmark symptom driving individuals to seek medical help^2^. Health-care expenditures and other consequences of painful knee OA, such as working disability, are considered to have a significant contribution to socio-economic burden^3^. So far there is no effective cure for OA. The determination of imaging biomarkers for painful and non-painful OA using several imaging modalities may help to define the disease phenotypes and subtypes more precisely and, therefore, initiate development of disease-modifying OA drugs and therapies^4^,^5^.

The aetiology of knee pain is heterogeneous and the relationship between structural changes, pain and function is not entirely clear^6^,^7^. The interplay of biological, psychologic and social factors may impact the OA pain experience of an individual^6^,^7^. Earlier studies aiming to explain the causes of pain using imaging methods have largely yielded inconsistent conclusions^6^,^7^,^8^,^9^,^10^. Discrepant results have been reported in a relationship with conventional radiography as the traditional diagnostic OA tool even after controlling for psycho-social factors^8^,^9^,^10^. Moreover, regardless of the high prevalence of pain in knee OA, several pathological changes were found on magnetic resonance images (MRI) in a large cohort of subjects without radiographic evidence of knee OA [Kellgren-Lawrence (KL) grade = 0]^11^,^12^. A recent study by Sharma *et al*.^12^ even demonstrated that in subjects with risk of OA, early MRI structural lesions are followed by incident radiographic OA and consequent symptoms^12^. Since the pain generation pathways in OA are not well understood, further research is required, including an assessment of the pain-structure relationship using relatively novel knee imaging techniques, *e.g*., ultrasound^6^,^13^.

In contrast to conventional radiography, ultrasound enables a direct assessment of changes in soft tissues. Furthermore, very recent studies suggest that it is more sensitive in the detection of osteophytes^14^,^15^,^16^. It also seems that early OA alterations can be detected in femoral cartilage using ultrasound, which is already depleted by more than 10% by the time the first knee changes can be seen on radiographs^13^,^17^. Femoral cartilage degeneration evaluated from still ultrasound images has been shown to associate with clinical symptoms and function^18^,^19^. Although the examinations of ultrasound-defined OA abnormalities have recently become more popular, especially the relationship between morphological changes and pain remains still vague^18^,^19^,^20^,^21^,^22^,^23^,^24^,^25^. Additionally, we believe that our developed wide-area ultrasound scanning technique may reveal even more OA changes than the traditional imaging of single predefined location(s) since it enables more comprehensive evaluation of joint tissues, particularly articular cartilage, osteophytes and menisci.

The aims of our study were: 1) to determine the prevalence of wide-area ultrasound scanned knee OA structural features in groups of symptomatic and asymptomatic subjects and 2) to determine the association of the structural features and their severity with pain and functional impairment.

## Materials and Methods

This study is part of the Oulu Knee Osteoarthritis (OKOA) study and was carried out between October 2012 and April 2014. Written informed consent was obtained from each participant. The study was performed in accordance with the Declaration of Helsinki and approved by the Ethical Committee of the Northern Ostrobothnia Hospital District, Oulu University Hospital.

### Symptomatic subjects

Eighty symptomatic subjects were selected from OA patients ranging in age between 30 and 70 years old (after initial examinations, the narrower age range of 45–70 was sought) in whom OA was suspected or diagnosed, or patients waiting for total knee arthroplasty at the Oulu University Hospital. The primary inclusion criteria were chronic knee pain and suspicion/diagnosis of knee OA or planned total knee arthroplasty. The subjects with previous significant knee joint trauma or surgery, existing inflammatory disease or other medical condition affecting the knee joint were excluded from the study. More details on the subject selection have been given in our earlier study^16^.

### Asymptomatic subjects

Eighty asymptomatic subjects were recruited from work colleagues, friends and family members of the research group or by advertisement in the local newspaper. The aim was to age- and gender- match the symptomatic group. Subjects were excluded if they have ever had repetitive or long-term (more than 2 weeks without interruption) pain in either knee, significant knee joint trauma or surgery, existing inflammatory disease or other medical condition affecting the knee joint, or significant overweight, *i.e.*, body mass index (BMI) > 35. Detailed subject selection is described in our earlier study^16^.

### Assessment of knee pain and function

Knee pain at the moment of filling in the questionnaire, further referred to as pain, and clinical symptoms and function over the past week were assessed using self-administered questionnaires. For assessment of pain, a visual analogue scale (VAS) ranging from 0–100 mm (0 meaning “no pain” and 100 meaning “very strong pain”) was used. For assessment of pain, stiffness and function over the past week, The Western Ontario and McMaster Universities Osteoarthritis Index (WOMAC) questionnaire (version with continuous VAS scales) was used^26^. The average value for each subscale and global WOMAC index as an average of the three subscales was calculated.

### Ultrasound imaging

**Figure 1:**
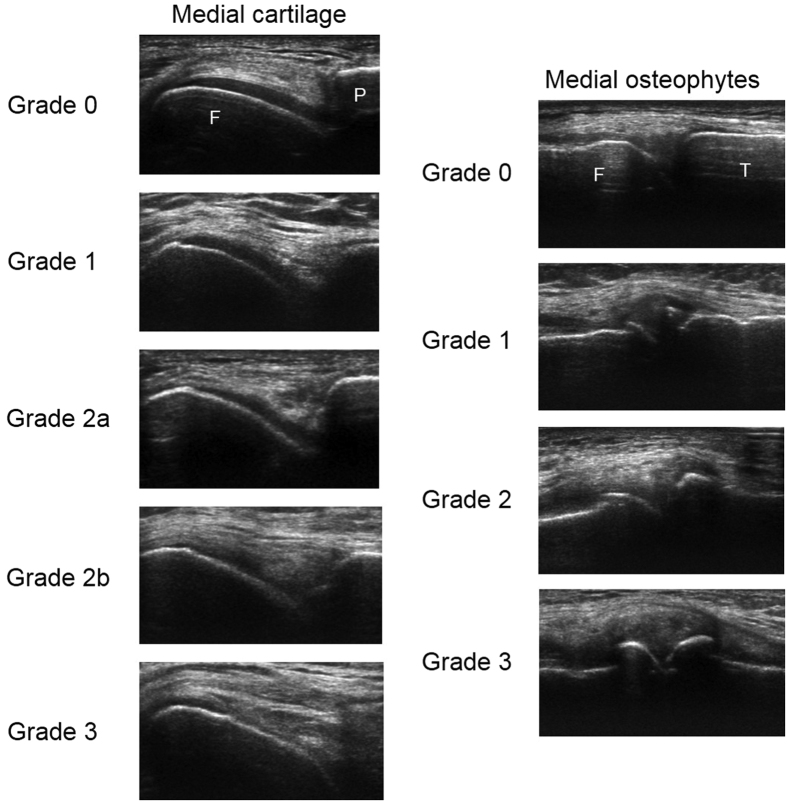
Example ultrasound images for semi-quantitative grading of medial femoral articular cartilage (left, F – femur, P – patella), and medial femoral and lateral osteophytes (right, F – femur, T – tibia). The definitions for articular cartilage degeneration grades^27^ assessed from transversal ultrasound images are as follows: Grade 0 – monotonous anechoic band with sharp hyperechoic anterior and posterior interfaces, Grade 1 – loss of the normal sharpness of cartilage interfaces and/or increased echogenicity of the cartilage, Grade 2a – in addition to the above changes, clear local thinning (less than 50%) of the cartilage, Grade 2b – Local thinning of the cartilage more than 50% but less than 100%, Grade 3–100% local loss of the cartilage tissue. The definitions for osteophyte grades^15^ assessed from longitudinal images are as follows: Grade 0 – no osteophyte, Grade 1 – small osteophyte, Grade 2 – medium osteophyte, Grade 3 – large osteophyte. In these images, the same grade osteophytes are always present on both femoral (F) and tibial (T) margin.

During ultrasound examination, the knee of the dominant hand side in asymptomatic subjects, and the more symptomatic knee or the knee going to be replaced in symptomatic subjects was imaged. If the subject had the same level of symptoms in both knees, the knee with the higher KL grade was examined. If the subjects had simultaneously the same level of symptoms and the same KL grade in both knees, the knee of the dominant hand side was examined. Commercially available ultrasound device (LOGIQ E9, GE Healthcare, Milwaukee, WI, USA) with 15 MHz linear transducer ML6–15 was used for the assessment. All settings except focus and image depth, were kept constant throughout the data collection. The imaging was performed by the trained sonographer (JP) who was also collecting all other data about the subjects. First, the subject was positioned supine with knee in full flexion and the flexion angle was measured by a goniometer. Medial, sulcus and lateral femoral articular cartilage was imaged in a transversal plane by proximal-distal probe sweeping over the anterior-central knee area, also including weight-bearing articular surfaces. The entire cartilage area reachable by ultrasound through the acoustic window was scanned. Then, the subject was asked to fully extend the knee and medial and lateral femoral and tibial osteophytes, and medial and lateral menisci were longitudinally imaged by anterior-posterior scanning from the medial and lateral side of the joint space. In each scanning session, 2–3 videos per region of interest were recorded in order to decrease the possible negative effect of operator-dependency and, hence, probability of false findings. The recorded videos from each site (depicting time below 10 s) were subsequently evaluated by the experienced rheumatologist (JMK), who was blinded to the clinical details and subject grouping, for cartilage degeneration and osteophyte presence and size using validated grading systems (Fig. 1)^15^,^27^. Cartilage was considered degenerated when loss of surface sharpness, increased inner echogenicity, local thinning and/or total loss of cartilage thickness were observed (Fig. 1). However, natural anatomical cartilage thinning at the femoral bone margins was considered to be normal. Meniscal extrusion was measured perpendicularly to the line connecting the tibial and femoral bone ends in mm. For comparisons of groups with and without meniscal extrusion, the pathological threshold was set to be equal or greater than 3 mm. The intra-rater agreement assessed earlier was moderate to nearly perfect (weighted kappa coefficient = 0.51–0.82) for cartilage and osteophyte grading and substantial to excellent (intra-class correlation coefficient = 0.72–0.91) for meniscal extrusion measurements^16^.

### Statistical analysis

Descriptive statistics of categorical variables were expressed as frequencies and percentages. Descriptive statistics of continuous variables were calculated as mean and standard deviation (SD) for normally distributed data, and median and range (min, max) for non-normally distributed data. The normality was examined by Kolmogorov-Smirnov test. The Student’s *t*-test or unequal variances *t*-test for normally distributed data, the Mann-Whitney U test for non-normally distributed data and the chi-square test for proportions were used to compare the differences between symptomatic and asymptomatic groups.

Negative binary regression modelling was used to estimate the associations of ultrasound-defined features with positively skewed outcome variables: pain severity, the WOMAC index and WOMAC subscales^28^. The associations of the following ultrasound-defined features were estimated in separate models: site-specifically assessed cartilage, osteophytes and meniscal extrusion; global, *i.e.*, maximum, femoral cartilage grade; maximum osteophyte grade in medial and lateral compartment, and global, *i.e*., maximum, osteophyte grade. The incidence rate ratios (IRRs) with a 95% confidence interval (CI) were estimated from the regression models to explain the associations. The IRR is interpreted as a ratio of VAS score in patients with an increased ultrasound grade relative to those with grade 0 (the reference group). The regression models were adjusted for age, gender and BMI and for global osteophyte grade and/or global femoral cartilage grade and/or medial and lateral meniscal extrusion depending on the analysed ultrasound-defined feature. Furthermore, we investigated the co-occurrence of global femoral cartilage degeneration and global osteophyte grade using Spearman’s correlation coefficient. The results were considered statistically significant when *p *<* *0.05. Statistical analyses were performed using commercial IBM SPSS software (ver. 22, SPSS Inc., Chicago, IL, USA).

## Results

### Study population

Out of 80 asymptomatic subjects 17 (21%) were excluded due to identified symptoms they did not report during the recruitment phase. One (1%) symptomatic subject was excluded due to missing ultrasound data. In total, 142 subjects were included in the study. The subjects’ characteristics are listed in Table 1. The mean age was 57.5 (SD 11.3) years, 61% were women and the mean BMI was 27.2 (SD 4.4) kg/m^2^. The symptomatic and asymptomatic groups were significantly different (*p *<* *0.001) in weight, BMI, knee flexion, pain and all WOMAC measures. The means and ranges of knee flexion angle for particular KL grades (0–4) in ascending order were: 117.5°(105.0°, 130.0°), 133.4° (109.0°, 148.0°), 130.8° (120.0°, 141.0°), 128.6° (115.0°, 147.0°) and 124.6° (112.0°, 141.0°). The number of subjects with particular KL grade can be found in Table 1. Note, that there were only two subjects with KL grade 0 in our study, and one of them was so symptomatic that he/she could not flex the knee much. For that simple reason, the mean flexion angle in the group of KL grade 0 is the lowest.

### Prevalence of ultrasound features

Any kind of cartilage changes were detected in 127 (89%) of subjects with 49 (39%) of them being asymptomatic (78% of the 63 asymptomatic subjects). Any kind of osteophytes were detected in 97 (68%) subjects, 28 (29%) of them were asymptomatic (44% of the 63 asymptomatic subjects). Eighty-seven (61%) subjects, 25 (29%) of them being asymptomatic (40% of the 63 asymptomatic subjects), had pathological meniscal extrusion in the medial site and 124 (87%), 55 (44%) of them being asymptomatic (87% of the 63 asymptomatic subjects) in the lateral site. The symptomatic and asymptomatic group differed (*p* < 0.001) in the presence of all features except lateral meniscal extrusion (Table 2).

### Association of ultrasound features with pain and function

The adjusted estimates of association of ultrasound-defined cartilage degeneration with pain and WOMAC index are presented in Table 3. The global femoral cartilage grade was strongly associated with pain and WOMAC index with the IRRs gradually increasing with higher ultrasound grade [IRR (95% CI) ranging from 5.1 (2.0, 13.0)–20.7 (5.0, 84.5) and 3.6 (1.4, 9.5)–9.6 (2.4, 38.1), respectively, when adjusted for all demographic variables, global osteophyte grade, and medial and lateral meniscal extrusions]. Site-specifically, especially cartilage thinning in sulcus and lateral site (grades ≥ 2a) was associated with pain (Table 3). Regarding WOMAC index the most prominent associations were found in medial site with IRR being the highest for grade 3, *i.e.*, local full thickness loss, [4.3 (1.1, 17.6), including all adjustments]. Subjects with grade 1 medial cartilage degeneration, determining the loss of sharpness of the cartilage interfaces and/or increased internal echogenicity, had statistically significant increase in IRR for pain and for the WOMAC index. No associations were observed in grade 1 sulcus nor grade 1 lateral cartilage degenerations (Table 3).

Presence of femoral, especially lateral, osteophytes revealed significantly increased IRRs for both outcomes pain as well as for WOMAC index (Table 4). All osteophyte grades in the medial femoral site were associated with worsened clinical symptoms (WOMAC index) during the past week (Table 4). Subjects with any lateral osteophytes reported increased pain [IRR (95% CI) ranging from 2.4 (1.3, 4.3) to 3.1 (1.3, 7.2)] and WOMAC index [IRR (95% CI) ranging from 2.2 (1.3, 3.9) to 2.7 (1.2, 6.1)], whereas only the presence of medium size (grade 2) osteophytes was associated with an increased WOMAC index in medial site. Having any osteophyte of any size and/or any meniscal extrusion did not increase either of the outcomes after all adjustments (Tables 4 and 5).

The results of associations of structural features with individual WOMAC subscales can be found as Supplementary Tables S1–S3. Global femoral cartilage grade had the highest IRRs in relation to each of the WOMAC subscales with stiffness being dominant especially in subjects with cartilage degeneration grade 3 in the medial site [IRR (95% CI) = 7.7 (2.1, 27.9)]. Moreover, the presence of femoral osteophytes was associated with all subscales.

The crude IRRs indicated gradual increase in pain and WOMAC index with more severe degeneration of all features except lateral meniscal extrusion in regard to WOMAC index (*p* = 0.056) and medial femoral osteophyte grade 2, in which the IRR slightly decreased [IRR (95% CI) = 3.2 (1.9, 5.5)] compared to grade 1 [3.4 (2.2, 5.3)], but remained significantly associated with increased pain.

### Co-occurrence of cartilage changes and osteophytes

A strong positive correlation [*r* (95% CI) = 0.78 (0.71, 0.84)] was found between global femoral cartilage and global osteophyte grades. The grade-wise co-occurrence of features is represented by cross-tabulation (Table 6).

## Discussion

In this study, the prevalence of wide-area scanned ultrasound-defined features, including femoral articular cartilage, osteophytes and meniscal extrusion, as well as their association with current pain and clinical symptoms and function during the past week were investigated. In the OKOA sub-population of symptomatic and asymptomatic subjects, degeneration of femoral cartilage was found to be the most prevalent feature and the strongest indicator (with the strongest degree-dependent association) of increased pain and disability. The presence of femoral lateral osteophytes was also associated with increased pain and disability of the subjects.

In OA, multiple joint tissues containing nociceptive fibres are the likely source of pain^6^. Although healthy cartilage is not a nociceptive tissue, nerve and vascular ingrowth is known to appear in degenerated cartilage^29^,^30^. Additionally, inflammation and subsequent synovial angiogenesis may develop as a secondary phenomenon due to interruption of the normal joint homeostasis by release of pro-inflammatory mediators, such as cytokines and matrix metalloproteinase, and degraded cartilage debris into a synovial cavity^31^,^32^. Ultimately, fibrillation of the superficial cartilage occurs along with the compositional alteration appearing as a loss of sharpness or clarity of a cartilage-soft tissue interface in ultrasound images^33^,^34^. Decreased clarity of a cartilage-soft tissue interface and increased severity of focal cartilage lesions were also detected in patients with painful knee in comparison to asymptomatic controls^35^. Consequently, we may suggest that early cartilage changes seen in ultrasound may contribute to pain in knee OA.

Although using different ultrasound imaging method as well as different semi-quantitative grading system, our results confirm the finding of Chen *et al*.^18^, who reported on the degree-dependent association of VAS pain with cartilage degeneration^18^. On the other hand, in the study of Malas *et al*.^24^, a significant positive relationship between WOMAC subscales and the severity of cartilage degradation was not observed in the group of symptomatic patients despite using the same grading system as in study of Chen *et al*.^18^,^24^. In earlier studies, changes in absolute cartilage thickness were also measured from ultrasound images but they did not show any association with increased pain^23^,^24^,^25^. In our study, we have not evaluated the cartilage absolute thickness as the normal cartilage thickness varies among individuals^36^,^37^. Only one study which assessed cartilage thinning as present or absent has found a positive association with the WOMAC index^19^. The strong association of severe cartilage loss, *i.e.*, grade 2b and 3, with pain and impaired function, may be explained by subchondral bone denudation and exposure of bone nociceptors^6^. Albeit in the first case there is still some cartilage left, it might mean that full thickness loss might still occur in another location not reachable by way of an ultrasound beam.

In our study, we observed increased symptoms in patients having osteophytes in the lateral joint site. Similarly, association of presence of global osteophytes with the WOMAC index and correlation of predominant morphological changes in lateral compartment including osteophytes with the KOOS symptom subscale have been reported^19^,^38^. However, in the latter case, no relationship with the pain subscale was found^38^. Likewise in cartilage, neovascularisation, which can be accompanied by sensory nerve growth, is found in developing osteophytes and thus might be linked to pain generation^39^. Bone-related pain may also be caused by osteophyte irritation of sensory nerve endings of the adjacent synovium^40^. Discordant results on osteophyte-pain association have been reported in MRI studies^41^,^42^. Yet, Torres *et al*.^41^ suggest that, based on multiple positive radiographic findings in earlier literature, the relationship between osteophytes and pain might, at least partially, be caused by confounding bone attrition and/or bone marrow lesions, believed to be one of the major sources for OA pain, which are not easily assessable by either X-ray and not at all by ultrasound^41^. OA onset commonly affects single-knee compartment with a medial site being more prevalent^43^. When we compared prevalence of medial pathologies (*i.e.*, cartilage and/or osteophytes) with lateral osteophytes (data not shown), we found out that 49 subjects (16 of them symptomatic) out of 70 subjects with no lateral osteophytes had already medial OA signs with dominant cartilage changes (100%). Moreover, 19 subjects (9 symptomatic) had progressing cartilage loss (grade 2a and 2b). Thus, the association of lateral osteophytes with clinical symptoms may be an actual reflection of already progressed, medially initiated knee OA.

Ultrasound studies investigating the impact of meniscal extrusion on pain and other clinical outcomes found either no or only weak associations^19^,^22^,^23^,^24^,^25^. In our study, medial meniscal extrusion was associated with pain and the WOMAC index when adjusted only for demographic confounders. After further adjustments by global femoral cartilage and global osteophyte grade, the association became non-significant, suggesting that in symptomatic subjects the extruded meniscus was just a coexistent OA feature to other painful and disabling pathological processes within the knee.

Although the prevalence of most of the OA features significantly prevailed in the symptomatic group, the frequent presence of OA abnormalities in asymptomatic subjects supports the existence of a non-painful knee OA phenotype among our study population. Additionally, the relatively wide confidence intervals, particularly in global femoral cartilage grade, may be due to the same reason. Interestingly, the same subject proportion of each group (87%) had laterally extruded meniscus. For that reason, we also decided to compare the measured extrusion values, which did not differ. Since there is no standardised way to measure the meniscal extrusion and not many ultrasound studies have investigated it in the lateral site, to define the threshold of normal and pathological displacement is challenging. In a study by Verdonk *et al*.^44^ the mean normal lateral extrusion in a group of ten subjects was 3.77 (SD = 1.76) mm^44^. Therefore, an elevation of the normal threshold may be advisable in the future.

In our study population, the presence and severity of the femoral cartilage degeneration and osteophyte formation were occurring mostly hand-in-hand, except for some subjects with no osteophytes in whom early but also progressed cartilage changes were already present. Similarly, subjects with grade 1 small osteophytes already had ongoing cartilage thinning with one subject even having full thickness loss locally. This may suggest that in part of the studied subjects, cartilage changes precede the osteophyte growth.

With the benefits of being cheap, safe, and widely available, providing also high-resolution, multi-planar, real-time, and dynamic imaging, ultrasound possesses a high potential in knee OA assessment among other imaging modalities. Moreover, we believe that our imaging approach brings more insight into structure-symptom relationship as we depict the entire area reachable by the ultrasound. Thus, the most severe site-specific pathology can be detected. However, to determine exactly how much larger areas can be scanned with our technique compared to static scans from single predefined locations may be a topic for future studies. Also, our cartilage grading system includes both evaluation of cartilage echogenicity and interface sharpness as an early sign of deterioration and cartilage thinning as a result of OA progression.

There were also some limitations of our study. First, we did not examine inflammatory signs of OA such as synovitis, effusion or synovial hypertrophy often being markers of painful knee^19^,^20^,^22^,^23^,^45^. However, our focus was on patients with chronic pain, not acute flares, which are most often the main sign of inflammatory processes. Moreover, inflammation may be both a primary, as well as secondary event in OA as discussed earlier, and it may further exacerbate the structural damage of the knee joint^30^. Furthermore, we did not investigate the possible impact of mechanical factors, such as knee alignment, which are thought to play an important role in pain etiology^46^, nor the impact of psycho-social determinants, such as pain catastrophising or social support, on the pain experience of the individuals^6^. Thus, whether the pain and symptom deterioration was more likely structure-casual, inflammatory, mechanical or psychological, or an interaction of all these factors, remains unrevealed in our studied population. Although subjects were not restricted in the use of analgesics, as it was considered unethical to completely restrict the use of painkillers, their symptoms still remained. However, it is possible that the actual self-reported pain level and function might have reached higher values when painkillers were not used. It is also notable that for ethical reasons (radiation dose concerns) knee radiographs could not be obtained from the asymptomatic subjects. We also did not image the posterior site of the femoral cartilage, and part of the lateral site of the femoral cartilage cannot be depicted by ultrasound due to the patellar shadow which is increased in subjects with severe OA due to the restriction on fully flexing the knee. Therefore, it is possible that more severe cartilage damage was present in one of these sites being the reason of pain and/or functional impairment. Finally, in order to achieve the best possible spatial resolution without the need to alter the ultrasound settings (such as frequency or gain) between the subjects, asymptomatic subjects with BMI below 30 were preferred at the recruitment. Our knee MRI coil (MRI has been also conducted in OKOA study) was also designed primarily for “standard-sized” knee, thus limiting the subject selection.

In conclusion, using our developed wide-area ultrasound scanning technique we were able to show that femoral cartilage changes were most prevalent and strongly associated with clinical symptoms also confirming findings of earlier reports. The presence of lateral femoral osteophytes, but not necessarily their size, was also associated with increased pain and disability of studied subjects. As pain genesis in OA is very complex, based on our results the true structure-symptom causality cannot be concluded. Therefore, further longitudinal studies investigating all possible ultrasound imaging aspects of knee OA and their interplay and relationship to clinical symptoms are recommended in the future.

## Additional Information

**How to cite this article:** Podlipská, J. *et al*. Structure-symptom relationship with wide-area ultrasound scanning of knee osteoarthritis. *Sci. Rep.* **7**, 44470; doi: 10.1038/srep44470 (2017).

**Publisher's note:** Springer Nature remains neutral with regard to jurisdictional claims in published maps and institutional affiliations.

## Supplementary Material

Supplementary Information

## Figures and Tables

**Table 1:** Characteristics of symptomatic and asymptomatic subjects.

Characteristic	Symptomatic (*n* = 79)	Asymptomatic (*n* = 63)	*p*	All subjects (*n* = 142)
Gender			0.836^*^	
Female, *n* (%)	49 (62.0)	38 (60.3)		87 (61.3)
Male, *n* (%)	30 (38.0)	25 (39.7)		55 (38.7)
Age (y)	62 (34, 70)	59 (24, 70)	0.118^¤^	61 (24, 70)
Female	62 (39, 70)	57 (26, 70)	0.116^¤^	61 (26, 70)
Male	60.5 (34, 70)^#^	61 (24, 70)^#^	0.531^¤^	61 (24, 70)^#^
Height (cm)	168 (153, 185)	168 (150, 198)	0.939^¤^	168 (150, 198)
Weight (kg)	82 (56, 118)	72 (47, 98)	<0.001^¤^	78.3 (47, 118)
BMI (kg/m^2^)	29.1 ± 4.3	24.8 ± 3.2	<0.001^†^	27.2 ± 4.4
Knee flexion (°)	129.2 ± 8.7	138.3 ± 6.1	<0.001^¥^	133.3 ± 8.9
Pain VAS (mm)	31 (0, 98)	0 (0, 10)^$^	<0.001^¤^	4 (0, 98)
WOMAC index (mm)	25 (0, 86)	0 (0, 6)^$^	<0.001^¤^	4.5 (0, 86)
WOMAC pain (mm)	33 (0, 94)	0 (0, 9)^$^	<0.001^¤^	5 (0, 94)
WOMAC stiffness (mm)	26 (0, 100)	0 (0, 9)^$^	<0.001^¤^	5 (0, 100)
WOMAC function (mm)	21 (0, 86)	0 (0, 6)^$^	<0.001^¤^	4 (0, 86)
KL grade, *n* (%)^§^				
0	2 (2.5)			
1	21 (26.6)			
2	19 (24.1)			
3	20 (25.3)			
4	17 (21.5)			
Use of analgesics, *n* (%)	68 (86.1)			
Paracetamol				
No use	32 (40.5)			
A few days a month	10 (12.7)			
A few times a week	24 (30.4)			
Daily	13 (16.5)			
NSAIDs				
No use	35 (44.3)			
A few days a month	19 (24.1)			
A few times a week	16 (20.3)			
Daily	9 (11.4)			
Weak opioids				
No use	62 (78.5)			
A few days a month	4 (5.1)			
A few times a week	8 (8.9)			
Daily	6 (7.6)			

If not indicated otherwise, the values are medians and ranges (min, max) for non-parametric tests and means ± standard deviations for parametric tests. BMI – body mass index, WOMAC – Western Ontario and McMaster Universities Osteoarthritis Index, VAS – visual analogue scale, NSAID – non-steroidal anti-inflammatory drug. *Chi-square test; ^¤^Mann-Whitney U test; ^†^Student *t*-test; ^¥^Unequal variance *t*-test. ^#^No statistical difference between male and female group (Mann-Whitney U test. *P* > 0.05). ^§^Kellgren-Lawrence (KL) grades given at the subject selection process. Radiographs were evaluated by an experienced rheumatologist who was blinded to any patient details. ^$^Note, that the VAS between 0–10 mm was tolerated within the asymptomatic group.

**Table 2:** Prevalence of knee ultrasound-defined features in symptomatic and asymptomatic subjects.

Ultrasound feature	All subjects (*n* = 142)	Symptomatic (*n* = 79)	Asymptomatic (*n* = 63)	*p*
	*n* (%)	*n* (%)	*n* (%)	
Cartilage				
Global femoral grade				<0.001^*^
0	15 (10.6)	1 (1.3)	14 (22.2)	
1	35 (24.6)	9 (11.4)	26 (41.3)	
2a	45 (31.7)	27 (34.2)	18 (28.6)	
2b	29 (20.4)	26 (32.9)	3 (4.8)	
3	18 (12.7)	16 (20.3)	2 (3.2)	
Osteophytes				
Global grade				<0.001^*^
0	45 (31.7)	10 (12.7)	35 (55.6)	
1	46 (32.4)	21 (26.6)	25 (39.7)	
2	23 (16.2)	21 (26.6)	2 (3.2)	
3	28 (19.7)	27 (34.2)	1 (1.6)	
Meniscal extrusion				
Medial				
<3 mm	55 (38.7)	17 (21.5)	38 (60.3)	<0.001^*^
≥3 mm	87 (61.3)	62 (78.5)	25 (39.7)	
Mean (SD), mm	4.06 (2.20)	5.04 (2.44)	2.84 (0.88)	
Median (min, max), mm	3.43 (0.93, 12.96)	4.54 (1.84, 12.96)	2.78 (0.93, 4.91)	<0.001^¤^
Lateral				
<3 mm	18 (12.7)	10 (12.7)	8 (12.7)	
≥3 mm	124 (87.3)	69 (87.3)	55 (87.3)	
Mean (SD), mm	4.67 (1.52)	4.68 (1.65)	4.65 (1.37)	
Median (min, max), mm	4.37 (1.48, 9.35)	4.29 (1.48, 9.35)	4.44 (2.04, 7.87)	0.730^¤^

Global femoral cartilage grade – maximum of medial, sulcus and lateral femoral articular cartilage grades. Global osteophyte grade – maximum of medial and lateral femoral and tibial osteophyte grades. SD – standard deviation. ^*^Chi-square test, symptomatic vs. asymptomatic. ^¤^Mann-Whitney U test, symptomatic vs. asymptomatic.

**Table 3:** Association of ultrasound-defined cartilage degeneration with pain and the WOMAC index presented as an incidence rate ratio (IRR) and 95% confidence interval (95% CI).

Cartilage	Grade	*n* (%)	Pain score (VAS)				WOMAC index			
			IRR^1^ (95% CI)	*p*	IRR^2^ (95% CI)	*p*	IRR^1^ (95% CI)	*p*	IRR^2^ (95% CI)	*p*
Medial	0	23 (16)		#		#		#		#
	1	48 (34)	2.7 (1.5, 4.8)	0.001	2.3 (1.2, 4.5)	0.013	3.2 (1.7, 6.1)	<0.001	2.5 (1.2, 5.3)	0.015
	2a	40 (28)	5.3 (2.9, 9.9)	<0.001	2.9 (1.3, 6.3)	0.006	5.9 (3.1, 11.5)	<0.001	3.1 (1.3, 7.2)	0.009
	2b	20 (14)	5.1 (2.5, 10.3)	<0.001	1.8 (0.6, 5.4)	0.289	8.0 (3.8, 17.1)	<0.001	2.8 (0.9, 8.7)	0.074
	3	11 (8)	12.4 (5.2, 29.5)	<0.001	3.5 (0.9, 13.7)	0.079	15.2 (6.0, 38.1)	<0.001	4.3 (1.1, 17.6)	0.041
Sulcus	0	37 (26)		#, §		#, §		#, §		#
	1	45 (32)	1.1 (0.6, 1.9)	0.778	1.0 (0.5, 1.7)	0.903	1.6 (0.9, 2.7)	0.127	1.4 (0.8, 2.6)	0.244
	2a	31 (22)	3.3 (1.9, 5.8)	<0.001	2.5 (1.4, 4.5)	0.003	3.6 (2.0, 6.4)	<0.001	2.6 (1.4, 4.7)	0.003
	2b	20 (14)	3.7 (2.0, 6.7)	<0.001	2.2 (1.1, 4.5)	0.026	4.3 (2.3, 8.0)	<0.001	2.7 (1.3, 5.5)	0.006
	3	9 (6)	4.6 (1.9, 10.7)	0.001	2.6 (1.0, 7.0)	0.056	5.0 (2.1, 11.9)	<0.001	2.9 (1.1, 7.5)	0.029
Lateral	0	58 (41)		#, §		#, §		#, §		#, §
	1	52 (37)	1.8 (1.2, 2.7)	0.005	1.2 (0.8, 1.9)	0.426	1.7 (1.1, 2.5)	0.017	1.0 (0.6, 1.6)	0.995
	2a	27 (19)	2.5 (1.5, 4.2)	0.001	1.8 (1.0, 3.3)	0.050	2.4 (1.4, 4.0)	0.001	1.5 (0.8, 2.8)	0.180
	2b	5 (4)	4.4 (1.8, 11.2)	0.002	3.3 (1.2, 8.9)	0.018	3.7 (1.5, 9.5)	0.006	2.6 (1.0, 7.0)	0.062
	3	0 (0)	—	—	—		—	—	—	—
Global femoral	0	15 (11)		#		#		#		#
	1	35 (25)	4.8 (1.9, 12.1)	0.001	5.1 (2.0, 13.0)	0.001	4.3 (1.7, 10.8)	0.002	3.6 (1.4, 9.5)	0.008
	2a	45 (32)	15.0 (5.8, 38.6)	<0.001	18.1 (5.6, 58.3)	<0.001	9.6 (3.8, 24.3)	<0.001	6.6 (2.1, 20.5)	0.001
	2b	29 (20)	23.5 (8.8, 62.7)	<0.001	20.9 (6.0, 72.5)	<0.001	19.3 (7.3, 51.3)	<0.001	9.6 (2.9, 31.9)	0.000
	3	18 (13)	27.3 (9.5, 78.4)	<0.001	20.7 (5.0, 84.5)	<0.001	23.0 (7.9, 67.1)	<0.001	9.6 (2.4, 38.1)	0.001

WOMAC – Western Ontario and McMaster Universities Osteoarthritis Index, VAS – visual analogue scale. ^1^Adjusted for gender, age and body mass index (BMI). ^2^Adjusted for gender, age, BMI, global osteophyte grade, and medial and lateral meniscal extrusion. ^#^BMI also significantly associated in the model (*p* < 0.05); ^§^age also significantly associated in the model (*p *<* *0.05).

**Table 4:** Association of ultrasound-defined osteophytes with pain and the WOMAC index presented as an incidence rate ratio (IRR) and 95% confidence interval (95% CI).

Osteophytes	Grade	*n* (%)	Pain score (VAS)				WOMAC index			
			IRR^1^ (95% CI)	*p*	IRR^2^ (95% CI)	*p*	IRR^1^ (95% CI)	*p*	IRR^2^ (95% CI)	*p*
Medial femoral	0	73 (51)		#, §		#		#, §		#
	1	31 (22)	3.7 (2.4, 5.7)	<0.001	2.1 (1.2, 3.8)	0.010	3.5 (2.2, 5.5)	<0.001	2.3 (1.3, 4.0)	0.004
	2	17 (12)	2.4 (1.4, 4.3)	0.002	1.5 (0.7, 3.4)	0.300	3.2 (1.8, 5.7)	<0.001	2.1 (1.0, 4.3)	0.049
	3	21 (15)	5.0 (3.0, 8.5)	<0.001	3.0 (1.0, 9.5)	0.057	5.6 (3.3, 9.6)	<0.001	3.4 (1.1, 10.0)	0.030
Lateral femoral	0	79 (56)		#, §		#		#, §		#
	1	25 (18)	3.3 (2.0, 5.3)	<0.001	2.3 (1.3, 4.0)	0.004	3.0 (1.9, 4.9)	<0.001	2.1 (1.2, 3.6)	0.006
	2	18 (13)	4.6 (2.7, 7.9)	<0.001	2.9 (1.5, 5.7)	0.002	4.5 (2.6, 7.7)	<0.001	2.6 (1.3, 5.1)	0.006
	3	20 (14)	4.2 (2.5, 7.0)	<0.001	2.7 (1.2, 6.1)	0.016	4.2 (2.5, 7.0)	<0.001	2.4 (1.1, 5.4)	0.030
Medial tibial	0	74 (52)		#, §		#		#, §		#
	1	41 (29)	1.7 (1.1, 2.5)	0.015	0.9 (0.5, 1.4)	0.553	1.6 (1.0, 2.3)	0.036	1.0 (0.6, 1.6)	0.936
	2	16 (11)	2.0 (1.2, 3.6)	0.014	1.1 (0.5, 2.4)	0.866	2.3 (1.3, 4.1)	0.004	1.5 (0.7, 3.1)	0.312
	3	11 (8)	4.9 (2.4, 9.9)	<0.001	1.9 (0.6, 5.9)	0.247	4.9 (2.4, 9.8)	<0.001	2.1 (0.7, 6.2)	0.175
Lateral tibial	0	93 (66)		#, §		#		#, §		#
	1	29 (20)	1.5 (1.0, 2.4)	0.080	1.1 (0.7, 1.9)	0.613	1.9 (1.2, 3.0)	0.009	1.3 (0.8, 2.2)	0.298
	2	10 (7)	2.1 (1.0, 4.2)	0.037	1.8 (0.8, 3.8)	0.140	1.6 (0.8, 3.3)	0.196	1.3 (0.6, 2.9)	0.493
	3	10 (7)	3.8 (1.9, 7.4)	<0.001	2.7 (1.0, 7.4)	0.051	3.3 (1.7, 6.5)	0.001	1.9 (0.7, 5.2)	0.220
Medial compartment	0	57 (40)		#, §		#		#, §		#
	1	44 (31)	2.3 (1.5, 3.5)	<0.001	0.9 (0.5, 1.6)	0.749	2.5 (1.6, 3.8)	<0.001	1.3 (0.7, 2.3)	0.395
	2	19 (13)	3.0 (1.7, 5.2)	<0.001	1.3 (0.6, 2.9)	0.499	4.1 (2.3, 7.1)	<0.001	2.1 (1.0, 4.4)	0.042
	3	22 (16)	4.7 (2.8, 8.0)	<0.001	1.8 (0.6, 5.8)	0.313	5.6 (3.2, 9.6)	<0.001	2.4 (0.8, 7.2)	0.118
Lateral compartment	0	70 (49)		#, §		#		#, §		#
	1	33 (23)	3.7 (2.3, 5.9)	<0.001	2.4 (1.3, 4.3)	0.004	3.3 (2.1, 5.4)	<0.001	2.2 (1.3, 3.9)	0.003
	2	19 (13)	5.2 (3.0, 8.8)	<0.001	3.1 (1.5, 6.4)	0.002	5.0 (2.9, 8.6)	<0.001	2.7 (1.4, 5.5)	0.005
	3	20 (14)	4.9 (2.9, 8.4)	<0.001	3.1 (1.3, 7.2)	0.010	4.8 (2.8, 8.3)	<0.001	2.7 (1.2, 6.1)	0.019
Global	0	45 (32)		#, §		#		#, §		#
	1	46 (32)	2.1 (1.3, 3.3)	0.002	0.6 (0.3, 1.3)	0.233	2.2 (1.4, 3.5)	0.001	1.1 (0.5, 2.1)	0.854
	2	23 (16)	4.4 (2.6, 7.6)	<0.001	1.1 (0.4, 2.6)	0.891	4.9 (2.8, 8.5)	<0.001	1.9 (0.9, 4.1)	0.109
	3	28 (20)	4.6 (2.7, 7.7)	<0.001	1.0 (0.4, 3.0)	0.959	5.2 (3.1, 8.8)	<0.001	1.7 (0.6, 4.5)	0.280

WOMAC – Western Ontario and McMaster Universities Osteoarthritis Index, VAS – visual analogue scale. ^1^Adjusted for gender, age and body mass index (BMI). ^2^Adjusted for gender, age, BMI, global femoral cartilage grade, and medial and lateral meniscal extrusion. ^#^BMI also significantly associated in the model (*p *<* *0.05); ^§^age also significantly associated in the model (*p* < 0.05).

**Table 5:** Association of ultrasound-defined meniscal extrusion with pain and the WOMAC index presented as an incidence rate ratio (IRR) and 95% confidence interval (95% CI).

Meniscal extrusion	*n* (%)	Pain score (VAS)				WOMAC index			
		IRR^1^ (95% CI)	*p*	IRR^2^ (95% CI)	*p*	IRR^1^ (95% CI)	*p*	IRR^2^ (95% CI)	*p*
Medial	142 (100)	1.3 (1.1, 1.4)	<0.001^#,§^	1.1 (0.9, 1.2)	0.362^#^	1.3 (1.2, 1.4)	<0.001^#,§^	1.1 (0.9, 1.2)	0.330^#^
Lateral	142 (100)	1.0 (0.9, 1.2)	0.467^#,§^	1.0 (0.9, 1.1)	0.767^#^	1.0 (0.9, 1.1)	0.816^#,§^	1.0 (0.8, 1.1)	0.556^#^

WOMAC – Western Ontario and McMaster Universities Osteoarthritis Index, VAS – visual analogue scale. ^1^Adjusted for gender, age and body mass index (BMI). ^2^Adjusted for gender, age, BMI, global osteophyte grade and global femoral cartilage. ^#^BMI also significantly associated in the model (*p* < 0.05); ^§^age also significantly associated in the model (*p* < 0.05).

**Table 6:** The cross-tabulation representing the co-occurrence of global femoral cartilage and osteophyte grades.

		Global osteophyte grade				Total
		0	1	2	3	
Global femoral cartilage grade	0	15	0	0	0	15
	1	21	13	1	0	35
	2a	7	27	8	3	45
	2b	2	5	11	11	29
	3	0	1	3	14	18
Total		45	46	23	28	142
