# Supplementary material for: Structure-symptom relationship with wide-area ultrasound scanning of knee osteoarthritis
Source: Sci Rep. 2017 Mar 15;7:44470. doi: 10.1038/srep44470 (PMC5353612; doi:10.1038/srep44470)
Supplement: Supplementary Information [file srep44470-s1.pdf]

Structure-symptom relationship with wide-area ultrasound scanning of knee osteoarthritis

[1]Jana Podlipská (jana.podlipska@oulu.fi); [2]Juhani M. Koski (f.koski@fimnet.fi); [3,4]Päivi Kaukinen (paivi.kaukinen@kuh.fi); [5,6,7] Marianne Haapea (marianne.haapea@oulu.fi); [1,5,6]Osmo Tervonen (osmo.tervonen@oulu.fi); [3,4]Jari P. Arokoski (jari.arokoski@kuh.fi); [1,5,6]Simo Saarakkala (simo.saarakkala@oulu.fi)

Supplementary Table S1: Association of ultrasound-defined cartilage degeneration with WOMAC subscales presented as an incidence rate ratio (IRR) and 95% confidence interval (95% CI)

			WOMAC pain				WOMAC stiffness				WOMAC function			
Cartilage	Grade	*n* (%)	IRR[1] (95% CI)	*p*	IRR[2] (95% CI)	*p*	IRR[1] (95% CI)	*p*	IRR[2] (95% CI)	*p*	IRR[1] (95% CI)	*p*	IRR[2] (95% CI)	*p*
Medial	0	23 (16)		#		#		#		#		#		#
	1	48 (34)	2.5 (1.3, 4.6)	0.004	2.0 (1.0, 4.1)	0.055	3.0 (1.7, 5.5)	< 0.001	2.8 (1.4, 5.7)	0.004	3.5 (1.8, 6.8)	< 0.001	2.6 (1.2, 5.7)	0.014
	2a	40 (28)	4.9 (2.6, 9.2)	< 0.001	2.6 (1.2, 5.8)	0.021	6.3 (3.4, 11.8)	< 0.001	3.3 (1.5, 7.4)	0.003	6.1 (3.1, 12.1)	< 0.001	3.0 (1.2, 7.3)	0.015
	2b	20 (14)	5.6 (2.7, 11.5)	< 0.001	1.8 (0.6, 5.5)	0.272	8.8 (4.3, 18.1)	< 0.001	3.8 (1.3, 11.1)	0.014	8.6 (3.9, 18.8)	< 0.001	2.8 (0.9, 9.1)	0.087
	3	11 (8)	10.6 (4.4, 25.3)	< 0.001	2.7 (0.7, 10.8)	0.149	22.8 (9.6, 54.1)	< 0.001	7.7 (2.1, 27.9)	0.002	15.4 (6.0, 39.8)	< 0.001	4.1 (1.0, 17.7)	0.057
Sulcus	0	37 (26)		#,§		#		#,§		#,§		#,§		#
	1	45 (32)	1.1 (0.6, 2.0)	0.701	1.1 (0.6, 1.9)	0.860	0.7 (0.4, 1.3)	0.278	0.6 (0.3, 1.1)	0.092	1.9 (1.1, 3.3)	0.025	1.7 (0.9, 3.0)	0.078
	2a	31 (22)	2.9 (1.6, 5.0)	< 0.001	2.1 (1.2, 3.8)	0.015	2.4 (1.3, 4.3)	0.003	1.4 (0.8, 2.7)	0.270	4.2 (2.3, 7.4)	< 0.001	3.0 (1.6, 5.5)	0.001
	2b	20 (14)	3.3 (1.8, 6.0)	< 0.001	2.2 (1.1, 4.4)	0.027	3.0 (1.6, 5.6)	0.001	1.5 (0.7, 3.0)	0.293	5.1 (2.7, 9.5)	< 0.001	3.2 (1.6, 6.6)	0.002
	3	9 (6)	3.3 (1.4, 7.7)	0.007	2.1 (0.8, 5.4)	0.131	4.2 (1.7, 10.2)	0.002	1.5 (0.6, 4.3)	0.402	6.0 (2.6, 14.1)	< 0.001	3.5 (1.4, 9.2)	0.010
Lateral	0	58 (41)		#,§		#,§		#,§		#,§		#,§		#,§
	1	52 (37)	1.5 (1.0, 2.3)	0.048	0.9 (0.6, 1.5)	0.713	1.7 (1.1, 2.5)	0.014	1.1 (0.7, 1.7)	0.638	1.7 (1.1, 2.6)	0.012	1.0 (0.6, 1.6)	0.992
	2a	27 (19)	1.8 (1.1, 3.1)	0.026	1.2 (0.6, 2.2)	0.595	2.3 (1.4, 3.9)	0.002	1.7 (1.0, 3.0)	0.072	2.6 (1.5, 4.4)	< 0.001	1.6 (0.9, 2.9)	0.128
	2b	5 (4)	2.8 (1.1, 7.1)	0.031	1.9 (0.7, 5.2)	0.200	5.9 (2.3, 14.9)	< 0.001	3.8 (1.4, 10.1)	0.009	3.8 (1.5, 9.7)	0.005	2.7 (1.0, 7.4)	0.051
	3	0 (0)	-	-	-	-	-	-	-	-	-	-	-	-
Global femoral	0	15 (11)		#		#		#		#		#		#
	1	35 (25)	4.0 (1.7, 9.7)	0.002	3.7 (1.5, 9.0)	0.005	3.7 (1.5, 9.3)	0.004	2.8 (1.1, 7.3)	0.030	3.8 (1.5, 9.5)	0.004	3.1 (1.2, 8.1)	0.019
	2a	45 (32)	10.4 (4.3, 25.0)	< 0.001	9.0 (2.9, 27.9)	< 0.001	11.7 (4.7, 29.0)	< 0.001	6.7 (2.3, 20.1)	0.001	7.6 (3.0, 19.3)	< 0.001	4.9 (1.6, 15.0)	0.006
	2b	29 (20)	17.5 (6.9, 44.4)	< 0.001	10.7 (3.3, 34.9)	< 0.001	26.2 (9.9, 69.8)	< 0.001	10.5 (3.3, 34.1)	0.000	16.2 (6.1, 42.6)	< 0.001	7.6 (2.3, 25.1)	0.001
	3	18 (13)	20.4 (7.4, 56.7)	< 0.001	10.0 (2.5, 39.0)	0.001	35.0 (12.4, 98.9)	< 0.001	11.7 (3.0, 44.8)	0.000	18.9 (6.5, 54.7)	< 0.001	7.5 (1.9, 29.4)	0.004

WOMAC – Western Ontario and McMaster Universities Osteoarthritis Index, VAS – visual analogue scale

[1] Adjusted for gender, age and body mass index (BMI).

[2] Adjusted for gender, age, BMI, global osteophyte grade, and medial and lateral meniscal extrusion.

BMI also significantly associated in the model ($p < 0.05$); § age also significantly associated in the model ($p < 0.05$)

Supplementary Table S2: Association of ultrasound-defined osteophytes with WOMAC subscales presented as an incidence rate ratio (IRR) and 95% confidence interval (95% CI)

Osteophytes	Grade	*n* (%)	WOMAC pain				WOMAC stiffness				WOMAC function			
			IRR[1] (95% CI)	*p*	IRR[2] (95% CI)	*p*	IRR[1] (95% CI)	*p*	IRR[2] (95% CI)	*p*	IRR[1] (95% CI)	*p*	IRR[2] (95% CI)	*p*
Medial femoral	0	73 (51)		#,§		#		#,§		#		#,§		#
	1	31 (22)	3.2 (2.1, 5.0)	< 0.001	2.0 (1.2, 3.5)	0.013	4.7 (3.0, 7.4)	< 0.001	2.8 (1.6, 4.8)	< 0.001	3.4 (2.2, 5.4)	< 0.001	2.3 (1.3, 4.1)	0.003
	2	17 (12)	3.0 (1.7, 5.3)	< 0.001	2.1 (1.0, 4.5)	0.043	4.4 (2.5, 7.7)	< 0.001	2.4 (1.1, 4.9)	0.022	3.2 (1.8, 5.6)	< 0.001	2.1 (1.0, 4.3)	0.054
	3	21 (15)	5.0 (2.9, 8.4)	< 0.001	3.7 (1.2, 11.3)	0.023	8.7 (5.1, 14.7)	< 0.001	3.0 (1.0, 9.2)	0.052	5.5 (3.2, 9.4)	< 0.001	3.3 (1.1, 9.8)	0.028
Lateral femoral	0	79 (56)		#,§		#		#,§		#		#,§		#
	1	25 (18)	2.8 (1.7, 4.5)	< 0.001	1.9 (1.1, 3.3)	0.014	4.3 (2.7, 7.0)	< 0.001	3.0 (1.0, 9.2)	< 0.001	3.0 (1.8, 4.9)	< 0.001	2.2 (1.3, 3.8)	0.005
	2	18 (13)	4.0 (2.4, 6.9)	< 0.001	2.6 (1.3, 5.0)	0.005	5.7 (3.4, 9.8)	< 0.001	2.4 (1.1, 4.9)	0.003	4.6 (2.7, 7.9)	< 0.001	2.7 (1.4, 5.4)	0.004
	3	20 (14)	3.7 (2.2, 6.2)	< 0.001	2.6 (1.2, 5.9)	0.018	6.6 (3.9, 11.3)	< 0.001	2.8 (1.6, 4.8)	0.027	4.1 (2.4, 6.9)	< 0.001	2.5 (1.1, 5.5)	0.026
Medial tibial	0	74 (52)		#,§		#		#,§		#		#,§		#
	1	41 (29)	1.7 (1.1, 2.5)	0.011	1.0 (0.6, 1.6)	0.939	1.3 (0.8, 1.9)	0.279	0.8 (0.5, 1.3)	0.403	1.5 (1.0, 2.3)	0.043	1.0 (0.6, 1.6)	0.974
	2	16 (11)	2.2 (1.2, 3.9)	0.008	1.2 (0.6, 2.6)	0.600	3.0 (1.7, 5.2)	< 0.001	2.2 (1.1, 4.4)	0.034	2.3 (1.3, 4.0)	0.005	1.5 (0.7, 3.1)	0.297
	3	11 (8)	4.4 (2.2, 8.8)	< 0.001	2.0 (0.7, 5.9)	0.199	6.9 (3.5, 13.9)	< 0.001	2.2 (0.8, 6.4)	0.136	4.7 (2.3, 9.6)	< 0.001	2.2 (0.7, 6.4)	0.166
Lateral tibial	0	93 (66)		#,§		#		#,§		#		#,§		#
	1	29 (20)	1.5 (0.9, 2.4)	0.087	1.1 (0.6, 1.8)	0.857	2.6 (1.6, 4.2)	< 0.001	1.7 (1.0, 2.9)	0.046	1.9 (1.2, 3.1)	0.006	1.4 (0.8, 2.3)	0.230
	2	10 (7)	1.5 (0.7, 3.0)	0.282	1.3 (0.6, 2.9)	0.481	1.9 (0.9, 3.9)	0.093	1.5 (0.7, 3.3)	0.339	1.6 (0.8, 3.3)	0.194	1.3 (0.6, 2.8)	0.519
	3	10 (7)	3.0 (1.5, 5.9)	0.002	2.1 (0.8, 6.1)	0.151	5.6 (2.8, 11.1)	< 0.001	2.3 (0.8, 6.6)	0.134	3.1 (1.6, 6.2)	0.001	1.8 (0.7, 4.9)	0.260
Medial compartment	0	57 (40)		#,§		#		#,§		#		#,§		#
	1	44 (31)	2.6 (1.7, 4.0)	< 0.001	1.2 (0.7, 2.2)	0.542	3.1 (2.0, 4.7)	< 0.001	1.5 (0.9, 2.6)	0.141	2.3 (1.5, 3.6)	< 0.001	1.3 (0.7, 2.3)	0.380
	2	19 (13)	3.8 (2.2, 6.5)	< 0.001	2.0 (0.9, 4.2)	0.075	6.1 (3.5, 10.7)	< 0.001	2.9 (1.4, 6.0)	0.003	3.9 (2.2, 6.8)	< 0.001	2.1 (1.0, 4.4)	0.042
	3	22 (16)	5.0 (3.0, 8.6)	< 0.001	2.5 (0.8, 8.0)	0.109	8.9 (5.2, 15.2)	< 0.001	2.6 (0.9, 8.2)	0.091	5.3 (3.1, 9.2)	< 0.001	2.4 (0.8, 7.0)	0.118
Lateral compartment	0	70 (49)		#,§		#		#,§		#		#,§		#
	1	33 (23)	2.8 (1.8, 4.5)	< 0.001	1.8 (1.0, 3.0)	0.040	4.7 (2.9, 7.5)	< 0.001	3.0 (1.7, 5.1)	< 0.001	3.5 (2.2, 5.7)	< 0.001	2.6 (1.5, 4.4)	0.001
	2	19 (13)	4.2 (2.4, 7.1)	< 0.001	2.4 (1.2, 4.8)	0.014	6.6 (3.8, 11.4)	< 0.001	2.9 (1.4, 5.8)	0.003	5.3 (3.1, 9.0)	< 0.001	3.1 (1.5, 6.2)	0.002
	3	20 (14)	4.1 (2.4, 7.0)	< 0.001	2.6 (1.1, 6.0)	0.026	7.8 (4.5, 13.3)	< 0.001	2.7 (1.2, 6.0)	0.017	4.8 (2.8, 8.3)	< 0.001	2.9 (1.3, 6.6)	0.010
Global	0	45 (32)		#,§		#		#,§		#		#,§		#
	1	46 (32)	2.3 (1.4, 3.6)	< 0.001	0.8 (0.4, 1.7)	0.562	2.6 (1.6, 4.1)	< 0.001	1.2 (0.6, 2.4)	0.528	2.1 (1.3, 3.3)	0.003	1.2 (0.6, 2.3)	0.664
	2	23 (16)	4.4 (2.6, 7.6)	< 0.001	1.5 (0.7, 3.3)	0.348	7.3 (4.2, 12.8)	< 0.001	2.5 (1.2, 5.4)	0.015	4.8 (2.8, 8.4)	< 0.001	2.1 (0.9, 4.5)	0.069
	3	28 (20)	4.8 (2.9, 8.2)	< 0.001	1.5 (0.5, 4.1)	0.435	8.0 (4.7, 13.6)	< 0.001	1.8 (0.7, 4.7)	0.220	5.0 (2.9, 8.5)	< 0.001	1.8 (0.7, 4.8)	0.209

WOMAC – Western Ontario and McMaster Universities Osteoarthritis Index, VAS – visual analogue scale

[1] Adjusted for gender, age and body mass index (BMI).

[2] Adjusted for gender, age, BMI, global femoral cartilage grade, and medial and lateral meniscal extrusion.

BMI also significantly associated in the model ($p < 0.05$); § age also significantly associated in the model ($p < 0.05$)

Supplementary Table S3: Association of ultrasound-defined meniscal extrusion with WOMAC subscales presented as an incidence rate ratio (IRR) and 95% confidence interval (95% CI)

Meniscal extrusion	*n* (%)	WOMAC pain				WOMAC stiffness				WOMAC function			
		IRR[1] (95% CI)	*p*	IRR[2] (95% CI)	*p*	IRR[1] (95% CI)	*p*	IRR[2] (95% CI)	*p*	IRR[1] (95% CI)	*p*	IRR[2] (95% CI)	*p*
Medial	142 (100)	1.3 (1.1, 1.4)	< 0.001[#,§]	1.1 (0.9, 1.2)	0.302[#]	1.4 (1.2, 1.5)	< 0.001[#,§]	1.1 (1.0, 1.3)	0.141[#]	1.3 (1.1, 1.4)	< 0.001[#,§]	1.1 (0.9, 1.2)	0.420[#]
Lateral	142 (100)	1.0 (0.9, 1.1)	0.506[#,§]	0.9 (0.8, 1.0)	0.190[#]	1.1 (1.0, 1.2)	0.102[#,§]	1.0 (0.9, 1.2)	0.499[#]	1.0 (0.9, 1.1)	0.765[#,§]	1.0 (0.8, 1.1)	0.542[#]

WOMAC – Western Ontario and McMaster Universities Osteoarthritis Index, VAS – visual analogue scale

[1] Adjusted for gender, age and body mass index (BMI).

[2] Adjusted for gender, age, BMI, global osteophyte grade and global femoral cartilage.

BMI also significantly associated in the model ($p < 0.05$); § age also significantly associated in the model ($p < 0.05$)
